# Risk Stratification Study of Indeterminate Thyroid Nodules with a next-generation Sequencing Assay with Residual ThinPrep® Material

**DOI:** 10.7150/jca.46086

**Published:** 2020-10-21

**Authors:** Huan Zhao, Weiwei Jing, Weihua Li, Zhihui Zhang, Jian Cao, Linlin Zhao, Yue Sun, Cong Wang, Yong Wang, Huiqin Guo

**Affiliations:** 1 Department of Pathology, National Cancer Center/ National Clinical Research Center for Cancer/Cancer Hospital, Chinese Academy of Medical Sciences and Peking Union Medical College.; 2 Department of Ultrasound, National Cancer Center/National Clinical Research Center for Cancer/Cancer Hospital, Chinese Academy of Medical Sciences and Peking Union Medical College.

**Keywords:** fine-needle aspiration, cytology, thyroid nodules, thyroid cancer, molecular diagnosis

## Abstract

**Objective:** The management of indeterminate thyroid nodules is challenging. Molecular testing has emerged as a promising method for stratifying this gray area of fine-needle aspiration (FNA) cytology. Next-generation sequencing (NGS) can be used to test a large variety of genetic changes with very small amounts of nucleic acids obtained from FNA samples.

**Methods:** Thyroid FNA assays were classified according to the Bethesda System for Reporting Thyroid Cytopathology after routine ThinPrep® slide preparation. Indeterminate nodules with surgical outcomes were assayed with an 18-gene NGS panel with the residual ThinPrep® material, including nodules categorized as atypia of undetermined significance (AUS)/follicular lesions of undetermined significance (FLUS) or follicular neoplasm (FN)/suspicious for a follicular neoplasm (SFN). We evaluated the diagnostic efficacy of the 18-gene panel for thyroid malignancies and potential malignancies and compared it with a well-accepted examination, ThyroSeq v2 testing.

**Results:** A total of 36 indeterminate nodules were assayed, seven were categorized as AUS/FLUS and 29 as FN/SFN. All of them had adequate DNA for the NGS procedure. When noninvasive follicular thyroid neoplasm with papillary-like nuclear features (NIFTP) was considered malignant, the risk of malignancy was 71.4% for AUS/FLUS nodules, and 69.0%for FN/SFN nodules. The 18-gene panel showed 72.0% sensitivity, 72.7% specificity, 85.7% positive predictive value (PPV), and 53.3% negative predictive value (NPV) in identifying malignancies and potential malignancies in the indeterminate nodules. Compared with a multicenter report from ThyroSeq v2 testing, 18-gene panel showed a lower NPV (*p*=0.005), but a higher PPV (*p*=0.02).

**Conclusions:** NGS assays are feasible on residual ThinPrep® material, with the advantage of not requiring additional FNA procedure. The 18-gene panel testing can be used as a 'rule in' test for surgical management based on indeterminate nodules and showed a lower NPV but a higher PPV compared to ThyroSeq v2 testing.

## Introduction

With the wide application of thyroid ultrasound in physical examinations, thyroid cancer has become the fastest growing type of cancer identified throughout the world, including on the Chinese mainland 1. Fine-needle aspiration (FNA) is the most effective diagnostic method for thyroid cancer. FNA allows the diagnosis of cancer or a benign nodule in most patients, although about 20% of FNA samples yield an indeterminate diagnosis 2. These indeterminate nodules include two subcategories of cytological diagnosis: atypia of undetermined significance (AUS)/follicular lesion of undetermined significance (FLUS) and follicular neoplasm (FN)/suspicious for a follicular neoplasm (SFN) 3. A predictor of indeterminate nodules that may place certain nodule types at higher malignancy rates is required.

Molecular testing is recommended by the 2015 American Thyroid Association guidelines as an adjunct technique to further stratify the risk of cytologically indeterminate nodules 4. To date, various commercial molecular tests, such as Afirma, ThyroSeq, or ThyGen X, have been approved by the U.S. Food and Drug Administration to evaluate cytologically indeterminate thyroid nodules 5. However, there is currently no molecular test that can definitively rule malignancy either in or out. More molecular data on indeterminate nodules are required. Next-generation sequencing (NGS) can be used to test a large variety of genetic changes simultaneously with a very small amount of nucleic acids, and allows multiple genes to be tested in FNA samples.

In this study, we used an 18-gene panel based on the NGS technology to test cytologically indeterminate nodules with residual ThinPrep® material. We evaluated the risk of malignancy in patients with a positive mutation detected with the 18-gene panel, and also compared the risk stratification achieved with the 18-gene panel to ThyroSeq v2 testing, which was also based on NGS platform and has been well-accepted.

## Materials and Methods

### Case selection

The thyroid FNA samples were collected from the National Cancer Center/National Clinical Research Center for Cancer/Cancer Hospital, Chinese Academy of Medical Sciences and Peking Union Medical College between November 2017 and June 2019 and analyzed retrospectively. The patients were selected on the basis of the following criteria: 1) cytological diagnosis of AUS/FLUS or FN/SFN according to the Bethesda System for Reporting Thyroid Cytopathology (TBSRTC) 3; 2) they had undergone thyroid surgery and had correlated cytological-histological results; and 3) an adequate residual specimen was available for DNA extraction after the routine cytological diagnosis. The cytological-histological correlation was performed by matching the locations and sizes of nodules in both the ultrasound and pathology reports. All the patients gave their informed consent before FNA. This study protocol was reviewed and approved by the Ethics Committee of the National Cancer Center/National Clinical Research Center for Cancer/Cancer Hospital.

### Specimen preparation

All FNA biopsies were performed under ultrasound guidance by radiologists. The aspirates were rinsed into a vial of CytoLyt^®^ (Hologic Inc., Marlborough, MA, USA) and prepared as slides with ThinPrep^®^ 2000 (Hologic Inc.). The slides were fixed in 95% alcohol and stained with Papanicolaou stain. They were then interpreted by two cytopathologists with experience ranging from 14 to 19 years. The residues were collected for DNA extraction. The residue selection criterion was defined as ten groups of cells on a slide in 10 ml of PreservCyt^®^ solution 6. The liquid materials were stored at -20 °C and used for molecular testing within 3 months.

### DNA extraction

After centrifugation, the cells were incubated in 500 μl of DNA lysis solution (1 mg/ml proteinase K, 10 mmol/l Tris-HCl (pH 8.0), 0.1 mol/l EDTA (pH 8.0), 0.5% (w/v) SDS) at 55 °C for approximately 12 h. The DNA was then extracted with the phenol-chloroform method and stored at -20 °C for future use. The concentration and purity of the DNA were measured with a NanoDrop ND-1000 (NanoDrop Technologies, Wilmington, DE, USA) spectrophotometer.

### Targeted DNA sequencing

Targeted DNA sequencing was performed for all patients with available DNA. The DNA was profiled with a capture-based targeted sequencing panel (Burning Rock Biotech, Guangzhou, People's Republic of China) that targets 18 genes (*BRAF*, *NRAS*, *HRAS*, *KRAS*, *RET*, *NTRK1*, *ETV6*, *ALK*, *PPARG*, *TERT*, *EIF1AX*, *PTEN*, *AKT1*, *PIK3CA*, *TP53*, *CTNNB1*, *TSHR*, and *GNAS*) and spans 140 kb of the human genome. In this way, we detected all single-nucleotide variants in these 18 genes and any gene fusions involving *RET*, *NTRK1*, *ETV6*, *ALK*, and *PPARG*. The design of 18-gene panel is based on data from public database and previous study in histology specimens 7,8. bioanalyzer high-sensitivity DNA assay was performed to assess the quality and size of the fragments. The available indexed samples were sequenced on a NextSeq 500 sequencer (Illumina Inc., San Diego, CA, USA]) as pair-end reads.

### Sequence data analysis

The sequence data were aligned to the human genome (hg19) with Burrows-Wheeler Aligner 0.7.10. Local alignment optimization and variant calling were performed with GATK v3.2-2. Both TopHat2 and Factera 1.4.3 were used for the DNA translocation analysis. To assess the level of DNA degradation, the insert size distribution and library complexity of each sample were computed. To avoid false positive mutation calls arising from DNA damage, different mutation calling thresholds were applied to DNA samples of different quality. Variants with population frequencies > 0.1% in the ExAC, 1000 Genomes, dbSNP, and ESP6500SI-V2 databases were grouped as common single-nucleotide polymorphisms and removed. Integrative Genomics Viewer (Broad Institute, USA) was used to visualize the variants aligned against the reference genome to confirm the accuracy of the variant calls by checking for possible strand bias and sequencing errors. Copy number variation was assessed by normalizing the read depth in each region to the total read number and region size, and correcting for GC bias using the LOESS algorithm.

### Statistical analysis

The cytological and molecular results were correlated with the histopathological results. A χ^2^ test was used to assess the differences in categorical variables. All statistical analyses were performed with SPSS 17.0, and *p* < 0.05 was considered statistically significant.

## Results

### Baseline characteristics of patients and nodules

Between November 2017 and June 2019, 434 thyroid nodules showed indeterminate cytology. Thirty-six indeterminate nodules with correlated surgical outcomes and residual ThinPrep® materials were enrolled. The patient and nodule characteristics are listed in **Table 1.** The mean age of patients was 49 years, and the ratio of females to males was 3:1. The median nodule size was 1.3 cm. Surgical pathology showed that 23 nodules were malignant, and the follicular variant of papillary thyroid carcinoma was the commonest malignant histopathology (47.8%). Benign pathologies included two adenomas and nine nodular hyperplasias. Two nodules were classified as noninvasive follicular thyroid neoplasm with papillary-like nuclear features (NIFTP).

### Risk of malignancy in indeterminate nodules

Among the 36 indeterminate nodules, seven were categorized as AUS/FLUS and 29 as FN/SFN. The ROM was 71.4% in the AUS/FLUS nodules and 69.0% in the FN/SFN nodules when NIFTP was considered malignant. If NIFTP was classified as benign, the prevalence of malignancy was 71.4% and 62.1%, respectively (**Table 2**).

### Diagnostic capacity of 18-gene panel and comparison with ThyroSeq v2 assay

**Figure 1:**
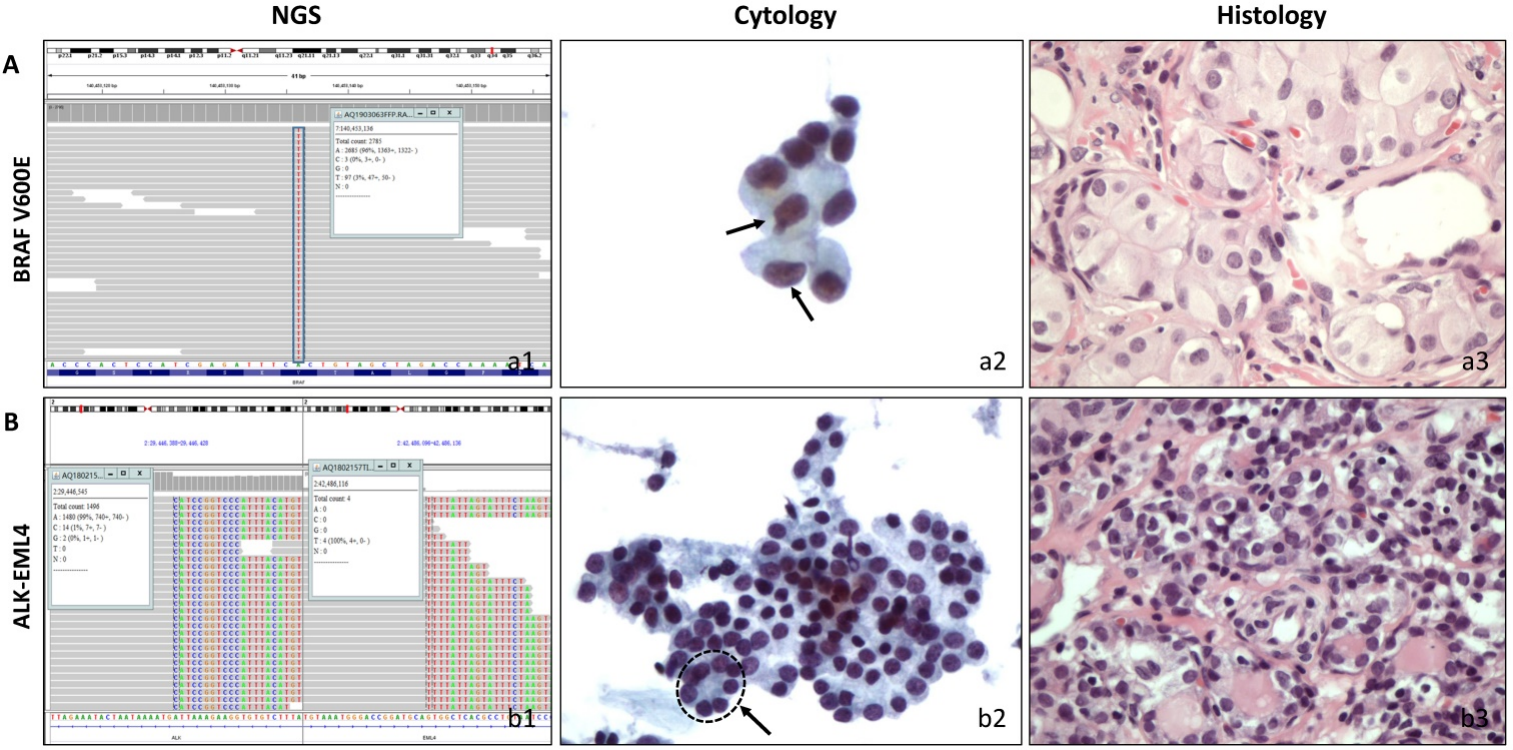
** Correlation of cytological and histological morphology and molecular results. A,** A follicular variant of papillary thyroid carcinoma with cytological diagnosis of AUS/FLUS. (a1) Molecular testing showed BRAF mutation. (a2) Cytopathology, a low cellular specimen composed of cells with enlarged nuclei. Two cells showed slightly irregular nuclei (arrow). The atypia cells were rare and insufficient for a diagnosis of suspicious malignant. (ThinPrep®, Papanicolaou stain, 400). (a3). Histopathology, thyroidectomy section (HE stain, 400). **B,** A follicular variant of thyroid papillary carcinoma with cytological diagnosis of FN/SFN. (b1) Molecular testing showed ALK/EML4 fusion. (b2) Cytopathology, a moderate cellular smear composed of uniform follicular cells with microfollicular arrangement (circle with arrow). (ThinPrep®, Papanicolaou stain, 400) (b3). Histopathology, thyroidectomy section (HE stain, 400).

The performance characteristics of the 18-gene panel testing for the diagnosis of thyroid malignancy including their sensitivity, specificity, positive predictive value (PPV), and negative predictive value (NPV) are shown in **Table 3.** When NIFTP was considered malignant, the 18-gene panel had 72.0% sensitivity, 72.7% specificity, 85.7% PPV, and 53.3% NPV. If NIFTP was considered benign, the PPV decreased to 76.2%, NPV maintained a value of 53.3%. **Table 3** also shows the comparison between our 18-gene panel and the previous reports from ThyroSeq v2. A statistical analysis was performed between present study and a multicenter report from ThyroSeq v2 in 2019 and 18-gene panel showed a lower NPV (*p*=0.005), but a higher PPV (*p*=0.02) with ThyroSeq v2. Two representative cases with positive molecular results are shown in **Figure 1.**

### Gene changes detected and the associated risk of malignancy

**Table 4** shows the gene changes detected with the 18-gene panel and the associated risk of malignancy. Among the gene mutations, the most commonly affected gene was *RAS (HRAS* Q61R*, NRAS* Q61K*,* Q61R, or G13R*)*, which was mutated in eight nodules, followed by *GNAS* (D448A, S455A, R844G, or A368T), which was mutated in three nodules (*GNAS* D448A and S455A were mutated simultaneously in one nodule). The identified gene fusions involved *RET* (*RET/NCOA4*) and *ALK* (*ALK*/*ELM4* or *ALK/CNTN4*) in two nodules, *ETV6* (*ETV6/NTRK3*) in three nodules. The *ALK*/*ELM4* and *ALK/CNTN4* fusions were encountered simultaneously in one NIFTP nodule. Another NIFTP nodule analyzed in our series carried the *NRAS* Q61R mutation. When NIFTP was considered malignant, mutations *BRAF*V600E,* HRAS* Q61R, *NRAS* G13R, the *TERT* promoter, *AKT1* E17K, *RET* R475W, *PIK3CA* H1047R, *CTNNBI* S33C, or *GNAS* (D448A, S455A, R844G or A368T) and a fusion of *RET/NCOA4*,* ALK*/*ELM4*, *ALK/CNTN4*or *ETV6/NTRK3* all carried a 100% risk of malignancy. However, mutations* NRAS* (Q61R, Q61K) and PTEN (A126D, K144) were not specifically associated with malignancy or potential malignancy. The malignancy rates (NIFTP considered malignant) for the *NRAS* and *PTEN* genes were 71% and 0%, respectively.

## Discussion

Over the past decade, molecular testing has emerged as a promising method for stratifying indeterminate thyroid FNAs. Several molecular testing panels, such as the Afirma Gene Expression Classifier, ThyroSeq v2, and ThyGenX/ThyraMIR, are commercially available in the USA 5, 9. However, none of them has been approved or is available in mainland China. Ultrasound characteristics and clinical features are currently the main criteria used to stratify indeterminate nodules. The decision to operate is rarely made with reference to molecular results.

We used NGS that targeted 18 genes to retrospectively analyze 36 cytologically indeterminate samples of thyroid lesions. All the samples were diagnosed as AUS/FLUS (7 cases) or FN/SFN (29 cases) with cytology, and the patients had undergone surgery based on their clinical and ultrasound features. The molecular analysis was performed after surgery with the residual liquid cytology samples after routine ThinPrep® slide preparation, which had been stored at -20 °C. To guarantee enough DNA for analysis, the selection criterion for the residues was defined as ten groups of cells on the slide in 10 ml of PreservCyt solution, as in our previous study 6. The DNA quality of all 36 samples fulfilled the requirements for NGS. Molecular testing based on residual ThinPrep® material has the advantage of not requiring an additional FNA.

In this study, the rates of malignancy for AUS/FLUS regardless of whether NIFTP was considered malignant or not were both 71.4%. The ROM of the FN/SFN nodules was 69.0% when NIFTP was considered malignant and fell to 62.1% when NIFTP was reclassified as benign. ROM was higher than the idealized ROMs described by TBSRTC 10. This discrepancy is mainly attributable to the surgical cohort selected. Our hospital is the national cancer center of China. The experiences of surgeons and radiologist make this selection more effective. The various differences in ROMs described in the present and previous studies emphasize the need for surgeons to understand their individual data, rather than rely on TBSRTC predictions 11-13.

When NIFTP was considered malignant, 18-gene panel showed 85.7% PPV and 53.3% NPV for the diagnosis of thyroid malignancy in indeterminate nodules. If NIFTP was considered benign, PPV decreased to 76.2%, NPV maintained a value of 53.3%. As previously reported, we considered NIFTP malignant when evaluating molecular tests in clinical practice because the recommended treatment fort NIFTP is surgical excision 14,15. Our 18-gene panel showed moderate NPV and high PPV when NIFTP was considered malignant, and may serve as a 'rule in' test for surgery.

Our 18-gene panel involves next-generation sequencing that detects gene mutations and fusions. The design of our panel is similar to a well-accepted test designated 'ThyroSeq v2', which was initially suggested to be both a “rule in” and “rule out” test because both its PPV (83%) and NPV (96%) were high 16. However, validation in the real world has suggested that its PPV may be lower than initially reported 17-19. Recently, a multicenter study reported a PPV of 59% and an NPV of 86% when NIFTP was considered malignant 19. Compared with that multicenter study, our 18-gene showed a lower NPV (*p* = 0.005), but a higher PPV (*p* = 0.02).

As in previous reports, *RAS* was the most frequent mutation identified in the indeterminate nodules in this study, and was not specifically associated with malignant or potentially malignant outcomes 20-22. Another false positive molecular result in our study was the mutation of* PTEN*. Two nodules with mutations in *PTEN* were both shown to be hyperplasia nodules. False *PTEN* mutations have rarely been reported in other studies 15, 17, 18, 23. This highlights the need for larger clinical studies to evaluate each mutation individually and to better characterize the risk of malignancy. The surgeon's familiarity with this information will allow more-appropriate clinical practices.

This study was not without limitations. Because residual ThinPrep® FNA samples were collected for molecular testing, more nodules categorized as AUS/FLUS were excluded than those categorized as FN/SFN because there were fewer cells in the AUS/FLUS residues. Fewer samples in AUS/FLUS subcategory than that in FN/SFN subcategory may have weakened the power to demonstrate the diagnostic capacities of the 18-gene test in AUS/FLUS nodules. The small sample size may have also limited our understanding of the malignant risk associated with specific gene changes.

Overall, residual ThinPrep® samples are suitable for NGS, and our 18-gene panel showed high PPV and moderate NPV for malignancy and potential malignancy. Therefore, it can be used as a 'rule in' test for stratifying indeterminate nodules. A lower NPV but a higher PPV was found with the use of 18-gene panel testing compared to the well-accepted ThyroSeq v2 testing.

## Figures and Tables

**Table 1:** Baseline characteristics of patients and nodules

Age, Mean (*SD*)	49 (13)
Sex ratio, F:M	3:1
Size (median)	1.3 cm
**Histopathological diagnosis of study population (n=36)**	
Benign (n=11)	11 (30.6%)
Nodular hyperplasia / with adenomatous hyperplasia	9 (81.8%)
Adenoma	2 (18.2%)
Malignant	23 (63.9%)
PTC-classical	1 (4.3%)
PTC-classial and follicular variants	2 (8.7%)
PTC-follicular variants	11 (47.8%)
PTC-cribriform-morular variants	1 (4.3%)
PTC-oncocytic variant	1 (4.3)
Follicular thyroid carcinoma	6 (26.1%)
Hürthle cell carcinoma	1 (4.3%)
NIFTP	2 (5.5%)

*SD*, standard deviation; NIFTP, non-invasive follicular thyroid neoplasm with papillary-like nuclear features; PTC, papillary thyroid carcinoma.

**Table 2:** Risk of malignancy and histopathology outcomes in indeterminate cytologic diagnoses

Cytologic diagnosis	Benign	NIFTP	Malignancy	ROM^a^	ROM^b^
AUS/FLUS	2	0	5	71.4%	71.4%
FN/SFN	9	2	18	69.0%	62.1%

AUS/FLUS, atypia of undetermined significance/follicular lesion of undetermined significance; FN/SFN, follicular neoplasm/suspicious for a follicular neoplasm; NIFTP, non-invasive follicular thyroid neoplasm with papillary-like nuclear features; ROM, risk of malignancy; ^a^ NIFTP considered malignant; ^b^ NIFTP considered benign.

**Table 3:** Comparison of published experiences with ThyroSeq v2 assay to the present study

Author	Panel	Material	Cytologic category	No. of surgery	Diagnostic performance	
Present study	18-gene	Residual liquid-based FNA sample	AUF/FLUS and FN/SFN	36	NIFTP = malignant	NIFTP = benign
					SN = 72.0%	SN = 69.6%
					SP = 72.7%	SP = 61.5%
					PPV = 85.7%	PPV = 76.2%
					NPV = 53.3%	NPV = 53.3%
Nikiforov [2014]	ThyroSeq v2	1dedicated FNA	FN/SFN	143	NIFTP = malignant	
					SN = 90%	
					SP = 93%	
					PPV = 83%	
					NPV = 96%	
Valderrabano [2017]	ThyroSeq v2	1 dedicated FNA	AUF/FLUS and FN/SFN	102	NIFTP = malignant	NIFTP = benign
					SN = 70%	SN = 73%
					SP = 77%	SP = 75%
					PPV = 42%	PPV = 33%
					NPV = 91%	NPV = 94%
Taye [2017]	ThyroSeq v2	1 dedicated FNA	AUS/FLUS and FN/SFN	60	NIFTP = malignant	
					SN = 89%	
					SP = 43%	
					PPV = 22%	
					NPV = 96%	
Marcadis [2019] (multicenter)	ThyroSeq v2	1 dedicated FNA	AUS/FLUS and FN/SFN	273	NIFTP = malignant	NIFTP = benign
					SN = 85%	SN = 87%
					SP = 62%	SP = 52%
					PPV = 59%	PPV = 35%
					NPV = 86%	NPV = 93%

AUS/FLUS, atypia of undetermined significance/follicular lesion of undetermined significance; FN/SFN, follicular neoplasm/suspicious for a follicular neoplasm; SN, sensitivity; SP, specificity; PPV, positive predictive value; NPV, negative predictive value; NIFTP, non-invasive follicular thyroid neoplasm with papillary-like nuclear features; FNA, fine needle aspiration.

**Table 4:** Detected gene changes and the associated risk of malignancy

Detected gene changes	Positive	ROM^a^	ROM^b^
**Mutations**			
*BRAF* V600E	2	100% (2/2)	100% (2/2)
*NRAS*	7	71% (5/7)	57% (4/7)
*NRAS* Q61K	3	67% (2/3)	67% (2/3)
*NRAS* Q61R	3	67% (2/3)	33% (1/3)
*NRAS* G13R	1	100% (1/1)	100% (1/1)
*HRAS*	1	100% (1/1)	100% (1/1)
*HRAS* Q61R	1	100% (1/1)	100% (1/1)
*TERT* promoter	1	100% (1/1)	100% (1/1)
*PTEN*	2	0% (0/2)	0% (0/2)
*PTEN* A126D	1	0% (0/1)	0% (0/1)
*PTEN* K144	1	0% (0/1)	0% (0/1)
*AKT1* E17K	1	100% (1/1)	100% (1/1)
*RET* R475W	2	100% (2/2)	100% (2/2)
*PIK3CA* H1047R	1	100% (1/1)	100% (1/1)
*CTNNB1* S33C	1	100% (1/1)	100% (1/1)
*GNAS*	4	100% (4/4)	100% (4/4)
*GNAS* D448A	1	100% (1/1)	100% (1/1)
*GNAS* S455A	1	100% (1/1)	100% (1/1)
*GNAS* R844G	1	100% (1/1)	100% (1/1)
*GNAS* A368T	1	100% (1/1)	100% (1/1)
**Fusions**			
*NCOA4/RET*	2	100% (2/2)	100% (2/2)
*ETV6/NTRK3*	3	100% (3/3)	100% (3/3)
*ELM4/ALK*	2	100% (2/2)	50% (1/2)
*CNTN4/ALK*	1	100% (1/1)	0% (0/1)

ROM, risk of malignancy; ^a^ NIFTP considered malignant; ^b^ NIFTP considered benign.

## Abbreviations

FNA: fine-needle aspiration
AUS: atypia of undetermined significance
FLUS: follicular lesions of undetermined significance
FN: follicular neoplasm
SFN: suspicious for a follicular neoplasm
NIFTP: noninvasive follicular thyroid neoplasms with papillary-like nuclear features
PPV: positive predictive value
NPV: negative predictive value
PTC: papillary thyroid carcinoma
TBSRTC: The Bethesda System for Reporting Thyroid Cytopathology
NGS: next-generation sequencing
